# Exacerbating Effects of Human Parvovirus B19 NS1 on Liver Fibrosis in NZB/W F1 Mice

**DOI:** 10.1371/journal.pone.0068393

**Published:** 2013-06-28

**Authors:** Tsai-Ching Hsu, Chun-Chou Tsai, Chun-Ching Chiu, Jeng-Dong Hsu, Bor-Show Tzang

**Affiliations:** 1 Institute of Microbiology and Immunology, Chung Shan Medical University, Taichung, Taiwan; 2 Clinical Laboratory, Chung Shan Medical University Hospital, Taichung, Taiwan; 3 Graduate Institute of Basic Medical Science, China Medical University, Taichung, Taiwan; 4 Department of Health and Nutrition Biotechnology, Asia University, Taichung, Taiwan; 5 Department of Neurology and Department of Medical Intensive Care Unit, Chunghua Christian Hospital, Chunghua, Taiwan; 6 Department of Pathology, School of Medicine, Chung Shan Medical University, Taichung, Taiwan; 7 Department of Pathology, Chung Shan Medical University Hospital, Taichung, Taiwan; 8 Department of Biochemistry, School of Medicine, Chung Shan Medical University, Taichung, Taiwan; 9 Institute of Biochemistry and Biotechnology, Chung Shan Medical University, Taichung, Taiwan; National Institute for Viral Disease Control and Prevention, CDC, China

## Abstract

Systemic lupus erythematosus (SLE) is an autoimmune disorder with unknown etiology that impacts various organs including liver. Recently, human parvovirus B19 (B19) is recognized to exacerbate SLE. However, the effects of B19 on liver in SLE are still unclear. Herein we aimed to investigate the effects of B19 on liver in NZB/W F1 mice by injecting subcutaneously with PBS, recombinant B19 NS1, VP1u or VP2, respectively. Our experimental results revealed that B19 NS1 protein significantly enhanced the TGF-β/Smad fibrotic signaling by increasing the expressions of TGF-β, Smad2/3, phosphorylated Smad2/3, Smad4 and Sp1. The consequent fibrosis-related proteins, PAI-1 and α-SMA, were also significantly induced in livers of NZB/W F1 mice receiving B19 NS1 protein. Accordingly, markedly increased collagen deposition was also observed in livers of NZB/W F1 mice receiving B19 NS1 protein. However, no significant difference was observed in livers of NZB/W F1 mice receiving B19 VP1u or VP2 as compared to the controls. These findings indicate that B19 NS1 plays a crucial role in exacerbating liver fibrosis in NZB/W F1 mice through enhancing the TGF-â/Smad fibrotic signaling.

## Introduction

Liver fibrosis is a dominant medical problem with significant morbidity and mortality [1]. The most commonly associated characteristic of fibrosis is excessive deposition of extracellular matrix (ECM) proteins, including glycoprotein, collagens and proteoglycan. The excess deposition of ECM proteins disrupts the normal architecture and functions of the liver [2]. Transforming growth factor β (TGF-β) has been recognized as a most potent fibrogenic cytokine, which stimulates the synthesis and deposition of ECM components [3]. After binding to the constitutively active type II receptor, TGF-β stimulates the Smad2/3 signaling by phosphorylating the type I receptor, which ultimately leads to liver cirrhosis, an end-stage consequence of fibrosis [1], [4]–[6].

Systemic lupus erythematosus (SLE) is an autoimmune disorder with unknown etiology [7] that impacts various organs including liver [8]. Increasing hepatic diseases are reported in SLE patients and recognized as important consequences of SLE, which also links to the pathogenesis of SLE [9]–[10]. Indeed, a previous study indicated that 11 SLE patients showed liver abnormality including fatty change, portal tract fibrosis, cellular infiltration, or even cirrhosis [11]. Another study of patients with SLE indicated that 124 of 206 patients tested had at least one abnormal result, and 43 met strict criteria for the existence of liver disease [12]. Similar results were also reported in lupus-prone animal models [13]–[15]. These findings strongly indicated the significant association of liver abnormality including fibrosis in SLE.

Human parvovirus B19 (B19) is known as an erythrovirus of human pathogen that consists a nonstructural protein (NS1) and two capsid proteins, VP1 and VP2 [16]. Recently, evidences have indicated that human parvovirus B19 may exacerbate or even induce SLE [17]–[19] and postulated a connection between these B19 viral proteins and the pathogenesis of SLE [20]–[24]. However, the effects of B19 viral proteins on liver fibrosis in SLE are still obscure. In the current study, we treated NZB/W F1 mice by injecting subcutaneously with recombinant B19 NS1, VP1u and VP2 proteins to investigate the effects of these B19 viral proteins on liver fibrosis in SLE.

## Materials and Methods

### Ethics

Animal experiments were approved by the Institutional Animal Care and Use Committee at Chung Shan Medical University.

### Preparation of recombinant B19 viral proteins

The recombinant human parvovirus B19 proteins were prepared as descried elsewhere [25]–[27]. Briefly, the cDNA of B19 VP1u were constructed onto pET-32a plasmid and transformed into E. coli (BL21-DE3). The recombinant B19 VP1u protein were then purified by Ni-NTA spin column (Qiagen, Chatsworth, CA) and spun through P50 and P30 Amicon (Millipore Billerica, MA) to avoid contaminative and degraded proteins [25]. The plasmid pQE40-NS1 containing nonstructural (NS1) gene of human parvovirus B19 was kindly provided by Professor Susanne Modrow, Institute for Medical Microbiology, Universität Regensburg, Regensburg, Germany. The NS1 protein was purified using Profinia denaturing IMAC purification kits and the Profinia protein purification system (Bio-Rad Laboratories, Inc. USA) according to the manufacturer's instructions [26]. The purified recombinant B19 NS1 and VP1u proteins were also analyzed by HPLC and the purities the three purified recombinant proteins were over 98%. The VP2 open reading frame (ORF) was obtained from the B19 genome (plasmid pYT104-C) by polymerase chain reaction using primers 5′-CGGAATTCCATGACTTCAGTTAATTCTGCAGAAGCC-3′ and 5′-GCGCGG CCGCTTACAATGGGTGCACACGGC-3′ containing *EcoR I* and *Not I* recognition sequences for subsequent cloning to pVL1393 baculoviral transfer vectors (Invitrogen). The constructed transfer vector and the BaculoGold DNA were used to co-transfect *Spodoptera frugiperda* (Sf9) cells by the calcium phosphate coprecipitation method according to the protocol provided by the manufacturer (PharMingen, San Diego, CA). Sf9 cells (Novagen, Merck, Germany) were maintained in Sf-900 II SFM (Invitrogen) in 100% room air at 28°C. Sf9 cells were infected with baculovirus stocks at a multiplicity of infection (MOI) of 5 and were harvested 72 h after infection. Recombinant VP2 proteins were purified by using 20% sucrose cushion and CsCl density gradient as described else there [28]. The yields of purified recombinant B19-NS1, -VP1u and VP2 proteins are 6.1, 15.6 and 17.7 mg/l, and the purities are nearly 96.8%, 98.1% and 97.8%, respectively.

### Animal and treatments

Twenty-four female NZB/W F1 mice at week 6 were purchased from Jackson Laboratories (Bar Harbor, ME, USA) and housed under supervision of the Institutional Animal Care and Use Committee at Chung Shan Medical University, Taichung, Taiwan. The animals were kept under a 12-h light-dark cycle and ambient temperature was maintained at 25°C. Animals were free access to water and standard laboratory chow (Lab Diet 5001; PMI Nutrition International Inc., Brentwood, MO, USA). Animal welfare and experimental procedures were performed according to the NIH Guide for the Care and Use of Laboratory Animals. All protocols were approved by the Institutional Animal Care and Use Committee of Chung Shan Medical University, Taichung, Taiwan. All the animals at the age of 8 weeks were randomly divided into 4 equal groups (6 mice each group) and injected subcutaneously with 20 ug purified B19-NS1, B19-VP1u, B19-VP2 recombinant proteins or phosphate-buffered saline (PBS) mixed 1∶1 (v/v) with Freund's complete adjuvant (Sigma-Aldrich, UK), respectively. The mice were boosted with the same dose mixed 1∶1 (v/v) with Freund's incomplete adjuvant (Sigma-Aldrich, UK) every two weeks for 3 times and then sacrificed at the age of 16 weeks by CO2 asphyxiation. The liver tissues were collected and stored at −80°C until use.

### Enzyme-linked immunosorbent assay (ELISA)

Detection of PAI-1 and TGF-β1 levels in serum was determined using a RayBio mouse PAI-1 EIA (Enzyme Immunoassay) kit (RayBiotech, Inc. Norcross, GA, USA) and an Abnova mouse TGF-β1 ELISA kit (Abnova, Walnut, Inc. CA, USA) according to manufacturers' instructions.

### Masson's trichrome staining

A small piece of liver tissue was collected and fixed in 10% buffered formalin. The sample was then embedded in paraffin, sliced into 5-µm-thick sections, and Masson's trichrome staining was performed, which stained collagen blue and myocytes red. Images were obtained using Zeiss Axiophot microscopes (Zeiss, Oberkochen, Germany).

### Preparation of tissue extract and determination of protein

All procedures were performed at 4°C in a cold room. The tissue samples obtained from NZB/W F1 mice were homogenized in 600 µl PRO-PREP™ solution (iNtRON Biotech, Korea) by 30 strokes using a Dounce Homogenizer (Knotes Glass, Vineland, NJ). The homogenates were centrifuged at 13,000 rpm for 10 min at 4°C and the supernatant was then stored at −80°C until use. Protein concentration of tissue extracts was determined according to the method described elsewhere [29] using bovine serum albumin as standards.

### Immunoblotting

Protein samples were separated in 10% or 12.5% SDS-PAGE and electrophoretically transferred to nitrocellulose membrane (Amersham Biosciences, Piscataway, NJ, USA) described elsewhere [30]. After blocking with 5% non-fat dry milk in (PBS), antibodies against transforming growth factor β (TGF-β), TGF-β receptor, Smad2/3, Smad4, phosphorylated Smad2/3 (p-Smad2/3), PAI-1, IL-6 and α-smooth muscle actin (SMA) (Santa Cruz Biotechnology, CA, USA) and β-actin (Upstates, Charlottesville, VA, USA) were diluted in PBS with 2.5% BSA and incubated for 1.5 h with gentle agitation at room temperature. The membranes were washed twice with PBS-Tween for 1 h and secondary antibody conjugated with horseradish peroxidase (HRP) (Santa Cruz Biotechnology, Santa Cruz, CA, USA) was added. Pierce's Supersignal West Dura HRP Detection Kit (Pierce Biotechnology Inc., Rockford, IL) was used to detect antigen–antibody complexes. Quantified results were performed by densitometry (Appraise, Beckman-Coulter, Brea, CA, USA).

### Statistical analysis

All of the statistical analyses were performed using SPSS 10.0 software (SPSS Inc., Chicago, IL). Three independent experiments were repeated. Statistical analyses were performed using the analysis of variance plus posterior multiple comparison test to test the difference. P<0.05 was considered statistically significant. The significant differences were stressed with symbols as shown in figures.

## Results

### B19 NS1 protein enhances the expressions of TGF-â1 and TGF-â1 receptor

**Figure 1:**
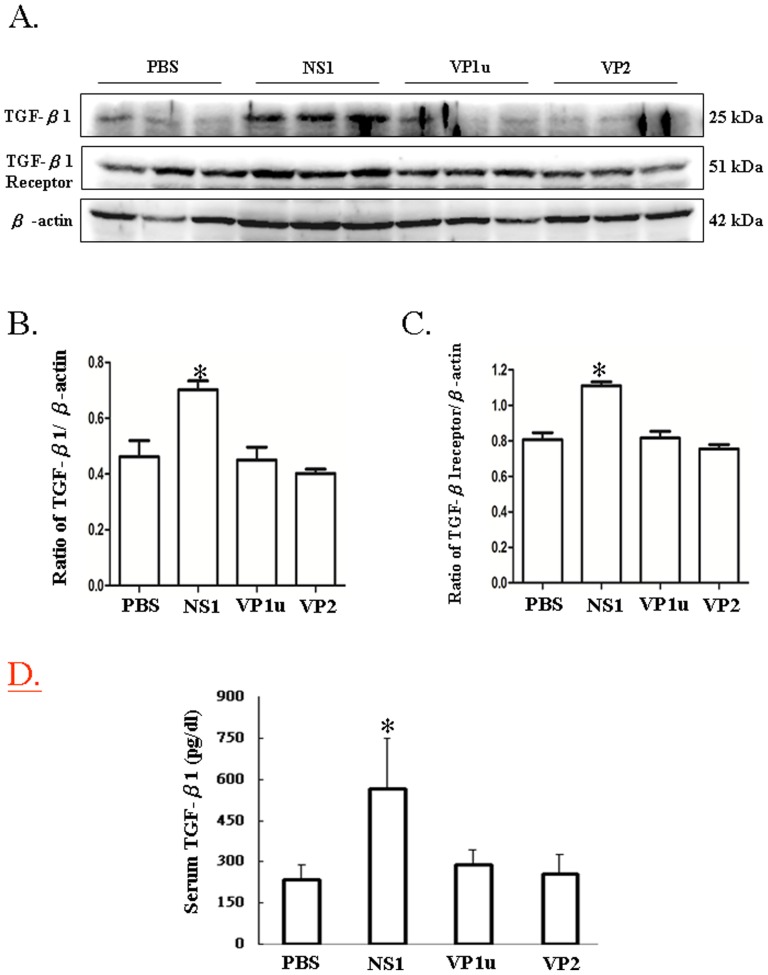
Expression of TGF-β1 and TGF-β1 receptor. Liver lysates obtained from the NZB/W F1 mice receiving PBS, NS1, VP1u or VP2 were probed with antibodies against (A) TGF-*β*1 and TGF-β1 receptor, respectively. Bars represent the relative protein quantification of (B) TGF-β1 and (C) TGF-β1 on the basis of β-actin. Serum level of (D) TGF-β1 was determined using an EIA kit. Similar results were observed in three independent experiments, and * indicates the significant difference, P<0.05.

To investigate the effects of human parvovirus B19 viral proteins on hepatic fibrosis, B19 NS1, VP1u and VP2 proteins were prepared as described in materials and methods and injected subcutaneously into NZB/W F1 mice. Notably, significantly increased expression of TGF-β1 protein was detected in liver of NZB/W F1 mice receiving B19 NS1 as compared to those mice receiving PBS (Fig 1A). In contrast, no significant variation on TGF-â1 expression in livers of NZB/W F1 mice receiving B19 VP1u or VP2 was detected as compared to those mice receiving PBS (Fig 1A). Similar results were also observed in expression of TGF-â1 receptor. Significantly increased TGF-â1 receptor was detected in livers of NZB/W F1mice receiving B19 NS1, whereas no significant variation of TGF-β1 receptor expression was observed in those mice receiving B19 VP1u or VP2 (Fig 1A). Quantified results and ratios of TGF-β1 and TGF-β1 receptor on the basis of β-actin were shown in figure 1B and 1C, respectively. Consistently, significantly increased level of serum TGF-β1 was detected in NZB/W F1mice receiving B19 NS1, whereas no significant variation of serum of TGF-β1 level was observed in those mice receiving B19 VP1u or VP2 (Fig 1D).

### B19 NS1 protein enhances the expression of Smad signaling

**Figure 2:**
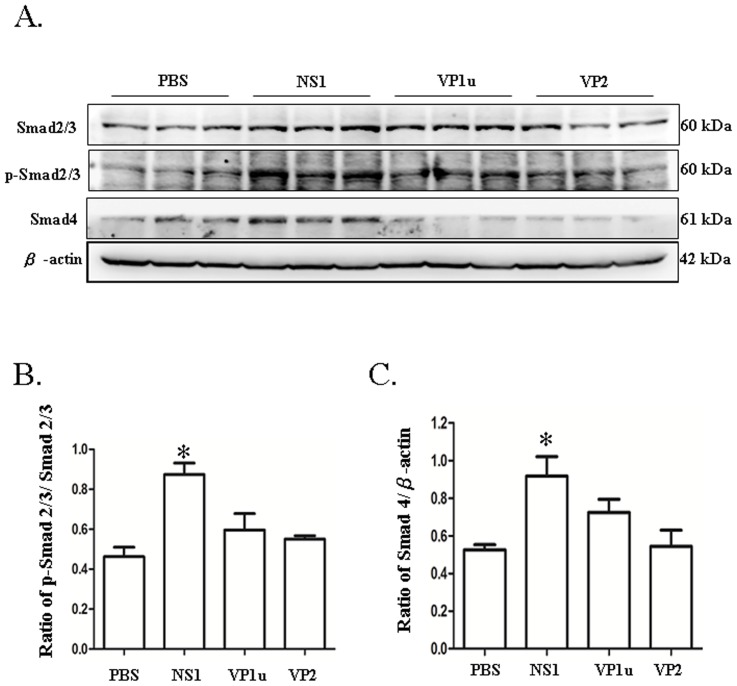
Expression of Smad2/3, phosphorylated Smad2/3 (p-Smad2/3) and Smad4. Liver lysates obtained from the NZB/W F1 mice receiving PBS, NS1u, VP1u or VP2 were probed with antibodies against (A) smad2/3, phosphorylated smad2/3 and smad4, respectively. Bars represent the relative protein quantification of (B) p-smad2/3/on the basis of Smad2/3 and (C) Smad4 on the basis of β-actin. Similar results were observed in three independent experiments, and * indicates the significant difference, P<0.05.

**Figure 3:**
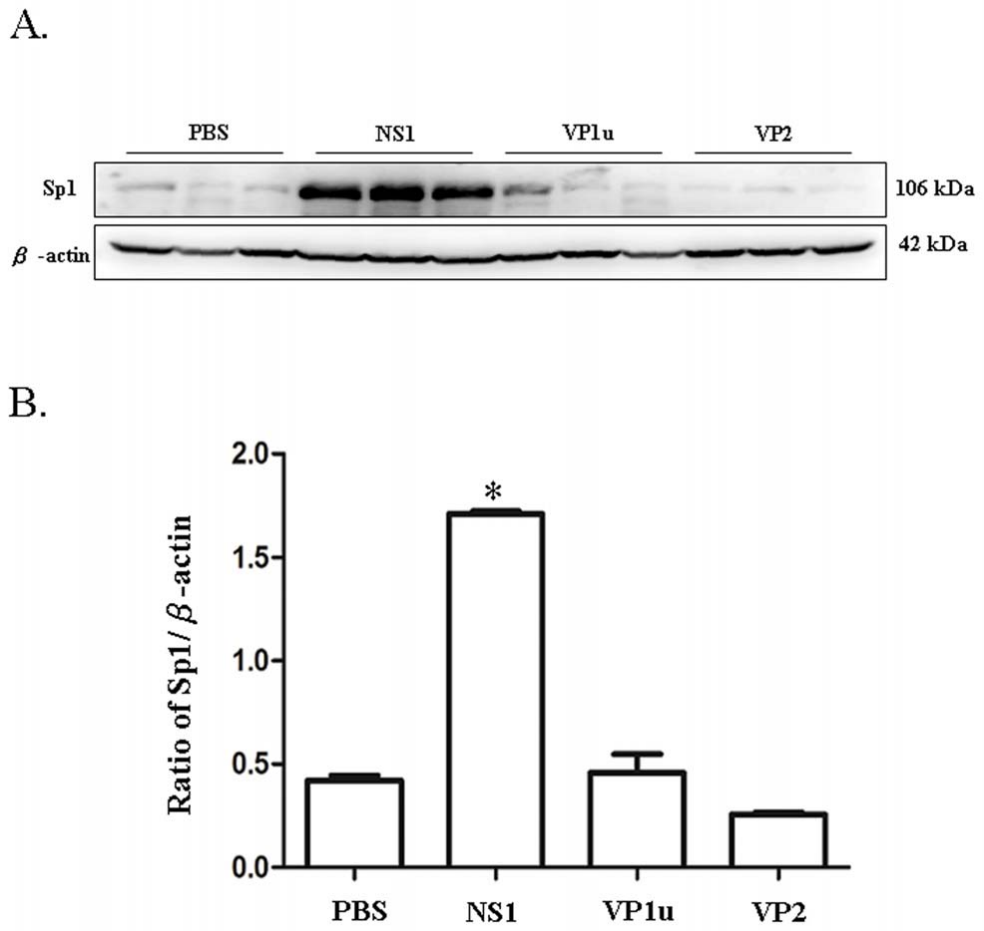
Expression of Sp1. Liver lysates obtained from the NZB/W F1 mice receiving PBS, NS1, VP1u or VP2 were probed with antibodies against (A) Sp1. Bars represent the relative protein quantification of (B) Sp1 on the basis of β-actin. Similar results were observed in three independent experiments, and * indicates the significant difference, P<0.05.

To further investigate the effects of human parvovirus B19 NS1 protein on hepatic fibrosis, the expression of Smad signaling were examined. Significantly increased ratio of relative protein quantification of p-Smad2/3/on the basis of Smad2/3 were observed in livers of NZB/W F1 mice receiving B19 NS1 protein as compared to those mice receiving PBS (Fig 2A). In addition, significantly increased Smad 4 protein was detected in livers of NZB/W F1 mice receiving B19 NS1 protein as compared to those mice receiving PBS (Fig 2A). In contrast, no significant variation on Smad4 expression and ratio of p-Smad2/3 on the basis of Smad2/3 were observed in livers of NZB/W F1 mice receiving B19 VP1u or VP2 as compared to those mice receiving PBS (Fig 2A). Quantified results were shown in figure 2B and 2C, respectively. Moreover, Sp1, a ubiquitous transcription factor that works in concert with Smad, were also examined. Significantly increased Sp1 protein was detected in livers of NZB/W F1mice receiving B19 NS1, whereas no significant variation of Sp1 expression was observed in those mice receiving B19 VP1u or VP2 (Fig 3A). Quantified results and ratio of Sp1 on the basis of â-actin were shown in figure 3B.

### B19 NS1 protein enhances the expression of PAI-1, á-SMA and collagen deposition

**Figure 4:**
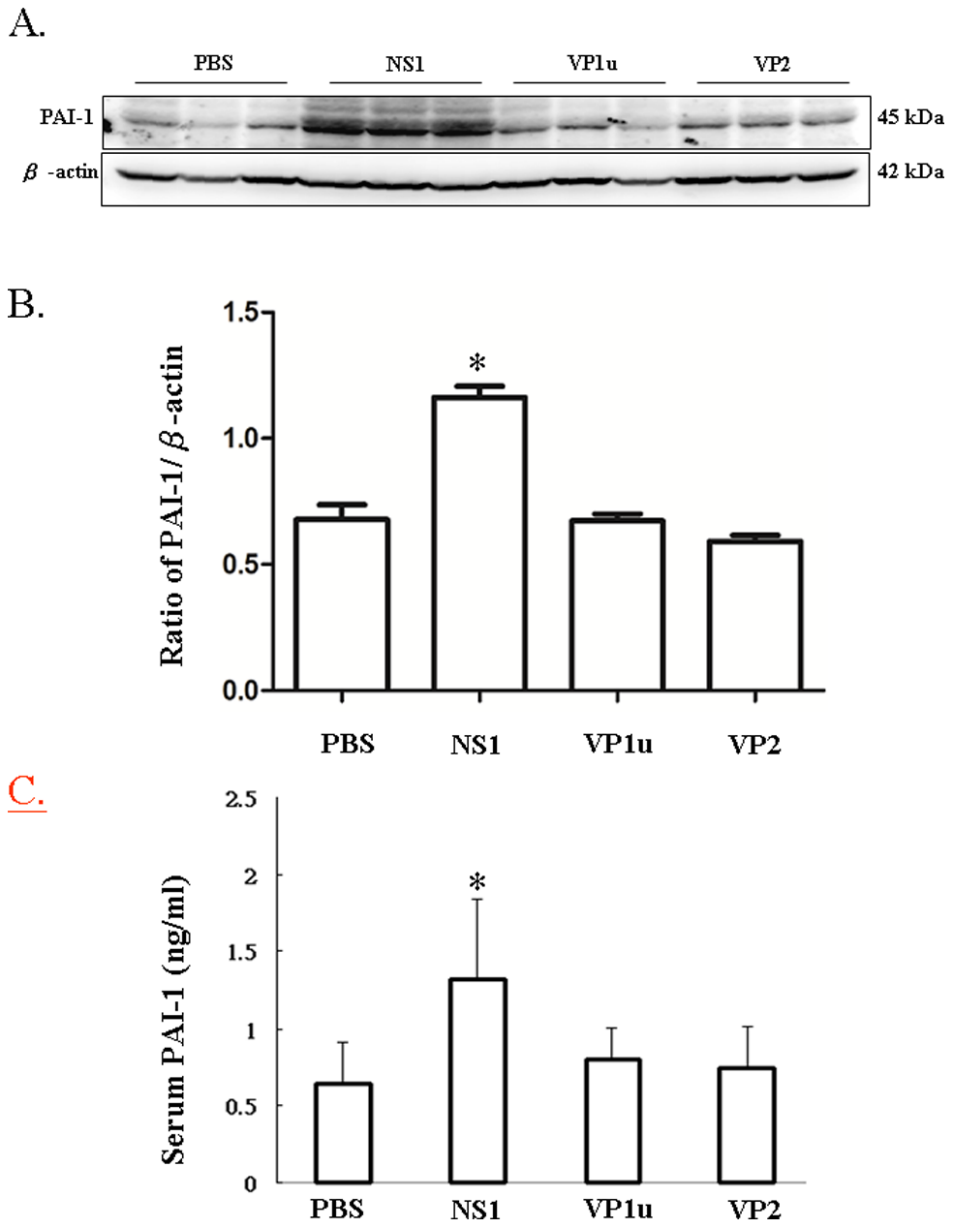
Expression of PAI-1. Liver lysates obtained from the NZB/W F1 mice receiving PBS, NS1, VP1u or VP2 were probed with antibodies against (A) PAI-1. Bars represent the relative protein quantification of (B) PAI-1 on the basis of β-actin. Serum level of (C) PAI-I was determined using an ELISA kit. Similar results were observed in three independent experiments, and * indicates the significant difference, P<0.05.

**Figure 5:**
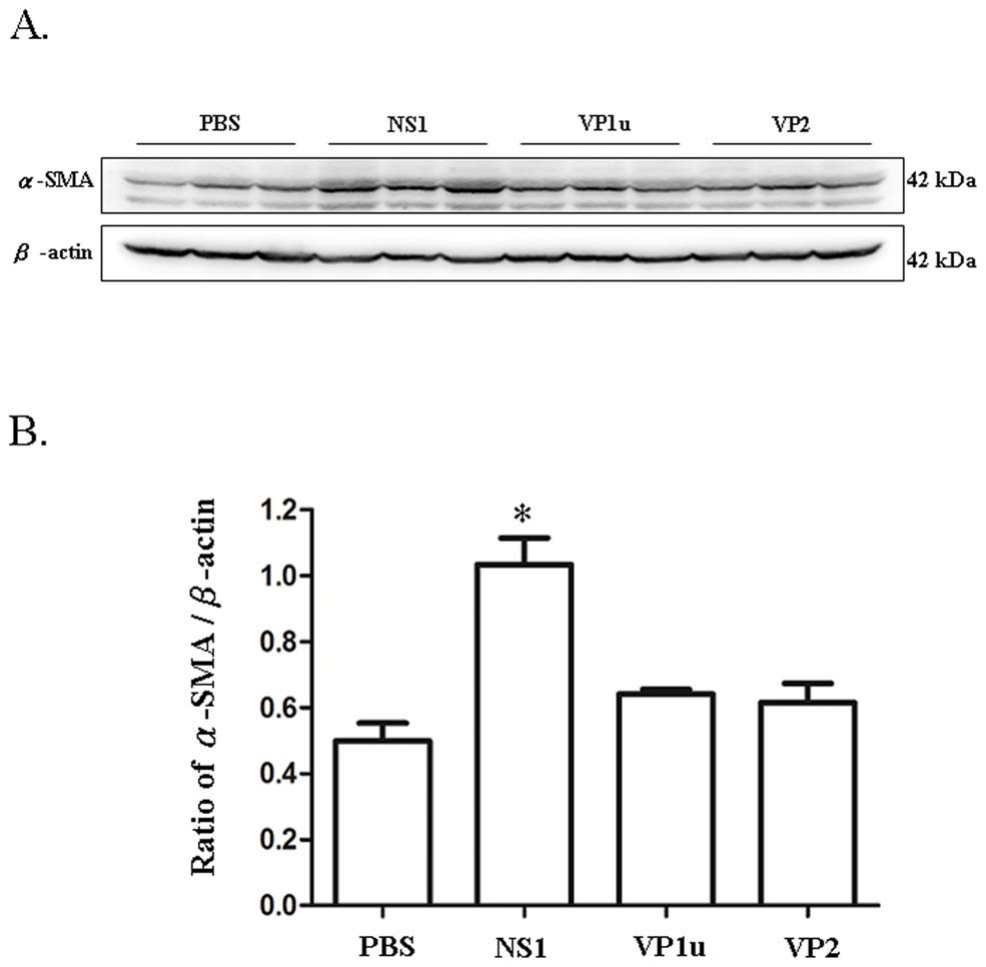
Expression of á-SMA. Liver lysates obtained from the NZB/W F1 mice receiving PBS, NS1, VP1u or VP2 were probed with antibodies against (A)á-SMA. Bars represent the relative protein quantification of (B)á-SMA on the basis of β-actin. Similar results were observed in three independent experiments, and * indicates the significant difference, P<0.05.

**Figure 6:**
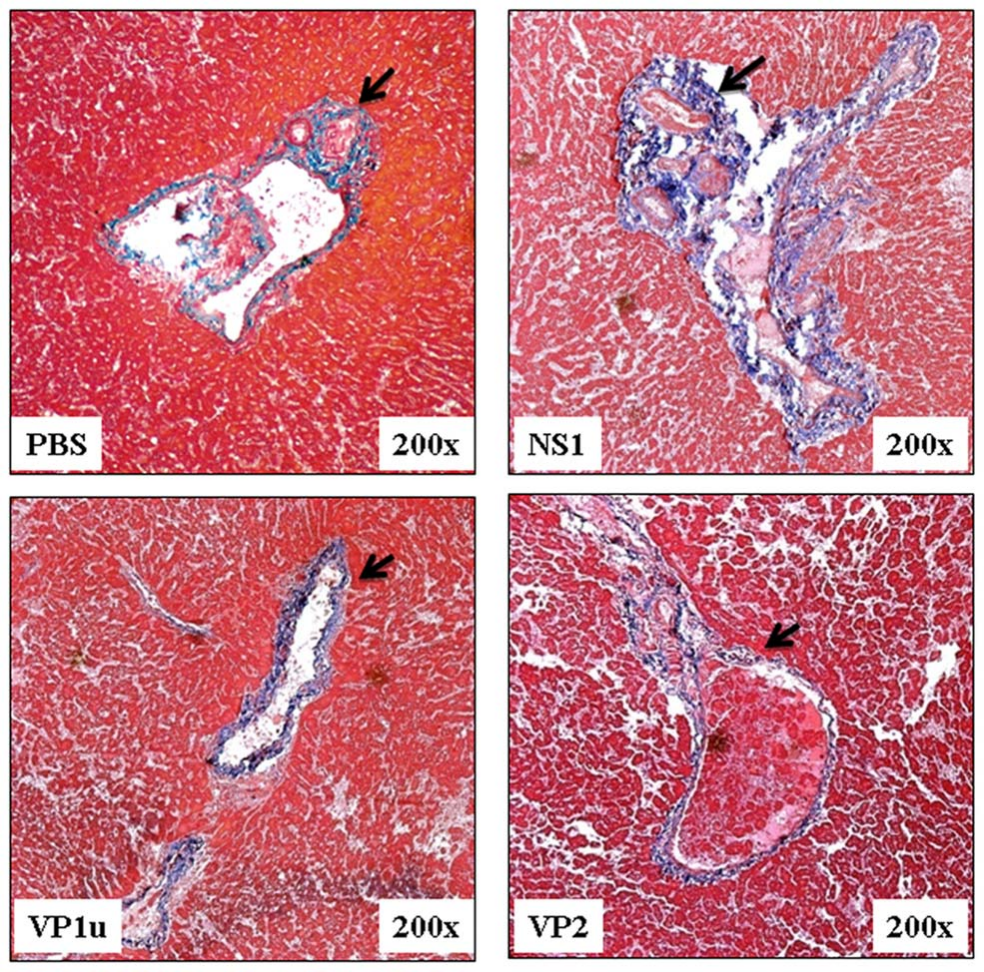
Representative photographs of cross-sections of liver with trichrome staining. Fibrosis area (blue) in liver section from NZB/W F1 mice receiving NS1 were revealed as compared to those mice receiving PBS, VP1u or VP2, respectively. (magnification, 200). Similar results were observed in three independent experiments.

Additionally, PAI-1, an inhibitor of fibrinolysis, was examined to further clarify the effect of B19 NS1 on hepatic fibrosis in NZB/W F1 mice. Significantly increased expression of PAI-1 protein was detected in livers and serum of NZB/W F1 mice receiving B19 N1 protein as compared to those mice receiving PBS (Fig 4A and 4C). Notably, no significant variation on PAI-1 level was observed in livers and serum of NZB/W F1 mice receiving B19 VP1u or VP2 as compared to those mice receiving PBS (Fig 4A and 4C). Quantified results of the Immunoblots were shown in figure 4B. Similar results was also observed in another fibrosis marker, á-SMA. Significantly increased expression ofá-SMA protein was detected in livers of NZB/W F1 mice receiving B19 N1 protein as compared to those mice receiving PBS (Fig 5A). Notably, no significant variation oná-SMA expression was detected in livers of NZB/W F1 mice receiving B19 VP1u or VP2 as compared to those mice receiving PBS (Fig 5A). Quantified results were shown in figure 5B. Meanwhile, markedly increased collagen deposition (blue area) was also detected in liver-section from NZB/W F1 mice receiving B19 NS1 as compared to those mice receiving PBS, B19 VP1u or VP2 (Fig 6), respectively.

## Discussion

Human parvovirus B19 is recognized as a trigger to exacerbate SLE [17]–[19]. Although B19 has been postulated to the pathogenesis of SLE in a variety of organs including liver [20]–[24], little is known about the effects of B19 viral proteins on hepatic fibrosis in SLE. In the present study, we firstly reported the aggravated effects of human parvovirus B19 NS1 protein on hepatic fibrosis in NZB/W F1 mice. Significant increases of TGF-β/Smad fibrotic signaling were detected in livers from NZB/W F1 mice receiving B19 NS1 protein via increasing the expressions of TGF-β, Smad2/3, p-Smad2/3, Smad4 and Sp1. Accordingly, the consequent fibrosis-related proteins, PAI-1 and α-SMA, were significantly induced in livers from NZB/W F1 mice receiving B19 NS1 protein. Meanwhile, markedly increased collagen deposition was also observed in livers from NZB/W F1 mice receiving B19-NS1 protein.

Increasing evidences have indicated the pivotal characters of various cytokines such as interleukin-6 (IL-6), TGF-β in the progression of hepatic fibrosis [31]–[32]. Indeed, a positive correlation between IL-6 expression in hepatocytes and the severity of hepatic fibrosis in patients with non-alcoholic fatty liver disease (NAFLD) was observed [33]. Another study also reported that polymorphisms induce high angiotensinogen and TGF-β1, the most abundant isoform of TGF-β, are associated with advanced hepatic fibrosis in patients with NAFLD [34]. Although a recent study of 12 clinical cases represented the associations of B19 infection with fibrotic diseases including idiopathic pulmonary fibrosis, scleroderma-associated pulmonary fibrosis, lymphocytic interstitial pneumonitis, and septal capillaritis [35], the effects of B19 on hepatic fibrosis in SLE are still obscure. B19 is an erythrovirus of human pathogen that consists a NS1 and two capsid proteins, VP1 and VP2 [16]. B19 NS1 protein is known to function as a transactivator of the B19 viral p6 and various cellular promoters, including those for the expression of tumor necrosis factor-α (TNF-α) and IL-6 [20], [36]–[37], which play crucial roles in pathogenesis of SLE [38]. Accordingly, our very recent study also revealed the aggravated effects of B19 NS1 protein on hepatic injury in NZB/W F1 mice by significantly enhancing the expression of inflammatory proteins including IL-6 (Fig S1) and MMP-9 through TNF-α/NF-κB (p65) signaling [27]. Since elevated TNF-α and IL-6 are also acknowledged to play crucial roles on hepatic fibrosis [31]–[32], these findings did provide a rational connection between B19 NS1 and liver fibrosis in SLE.

**Figure 7:**
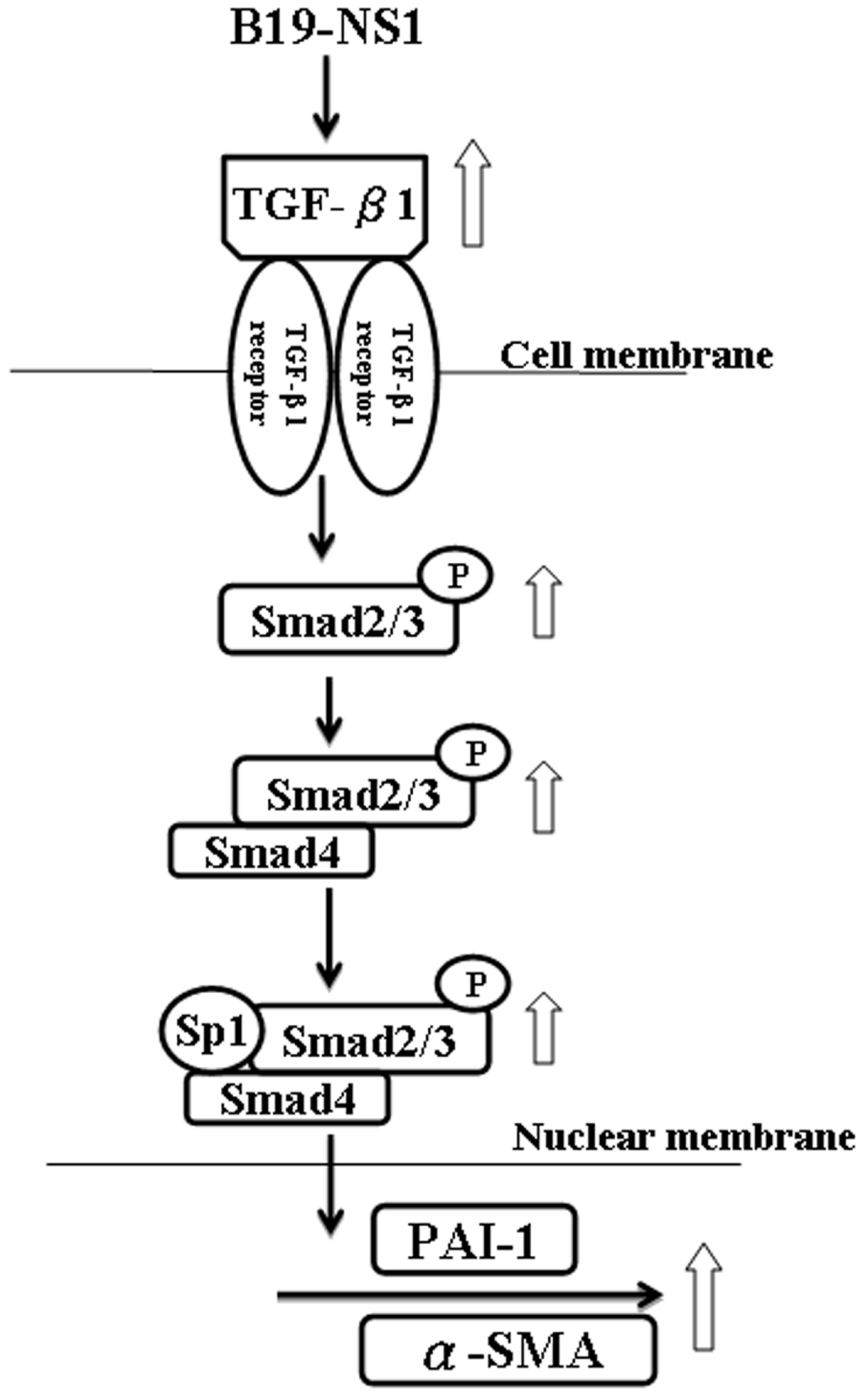
Schematic illustration of the molecular mechanisms involved in the B19 NS1-induced hepatic fibrosis. B19 NS1 increases the expression of TGF-β isoforms, which elicit late activation of Smad2/3 signaling. Activated Smad2/3 cooperates with Smad4 and Sp1 to induce the expressions of fiboriss related proteins, including PAI-1 and smooth-muscle-specific (á-SMA).

As illustrated in figure seven, B19 NS1 protein aggravates the hepatic fibrosis by enhancing the expression of TGF-β isoforms and activation of Smad2/3, which urge phosphorylated Smad2/3 to cooperate with Smad4 and Sp1 and induce the consequent fiboriss-related proteins, including PAI-1 and α-SMA (Fig 7). Taken together, this study firstly demonstrated an aggravated effects of B19 NS1 protein on hepatic fibrosis in NZB/W F1 mice through enhancing the TGF-β/Smad fibrotic signaling and could provide a possible explanation in exacerbating the hepatic fibrosis in SLE patients with B19 infection.

## Supporting Information

Figure S1 Expression of IL-6. Liver lysates obtained from the NZB/W F1 mice receiving PBS, NS1, VP1u or VP2 were probed with antibodies against (A) IL-6. Bars represent the relative protein quantification of (B) Sp1 on the basis of β-actin. Similar results were observed in three independent experiments, and * indicates the significant difference, P<0.05.
(TIF)
