# Correction: Exacerbating Effects of Human Parvovirus B19 NS1 on Liver Fibrosis in NZB/W F1 Mice

**DOI:** 10.1371/annotation/70d7eabb-a2dc-49c4-bd9d-e88b08cb2be9

**Published:** 2014-01-15

**Authors:** Tsai-Ching Hsu, Chun-Chou Tsai, Chun-Ching Chiu, Jeng-Dong Hsu, Bor-Show Tzang

The affiliations for the first and last authors are incorrect. The first author, Tsai-Ching Hsu is not affiliated with institutions 3 and 4 (as numerated in the PDF), but rather only institutions 1 and 2. The last author, Bor-Show Tzang, is not affiliated with institution 4 (as numerated in the PDF), but rather only with institutions 2, 8 and 9.

