# Supplementary figures and images for: Exacerbating Effects of Human Parvovirus B19 NS1 on Liver Fibrosis in NZB/W F1 Mice
Source: PLoS One. 2013 Jun 28;8(6):e68393. doi: 10.1371/journal.pone.0068393 (PMC3695896; doi:10.1371/journal.pone.0068393)

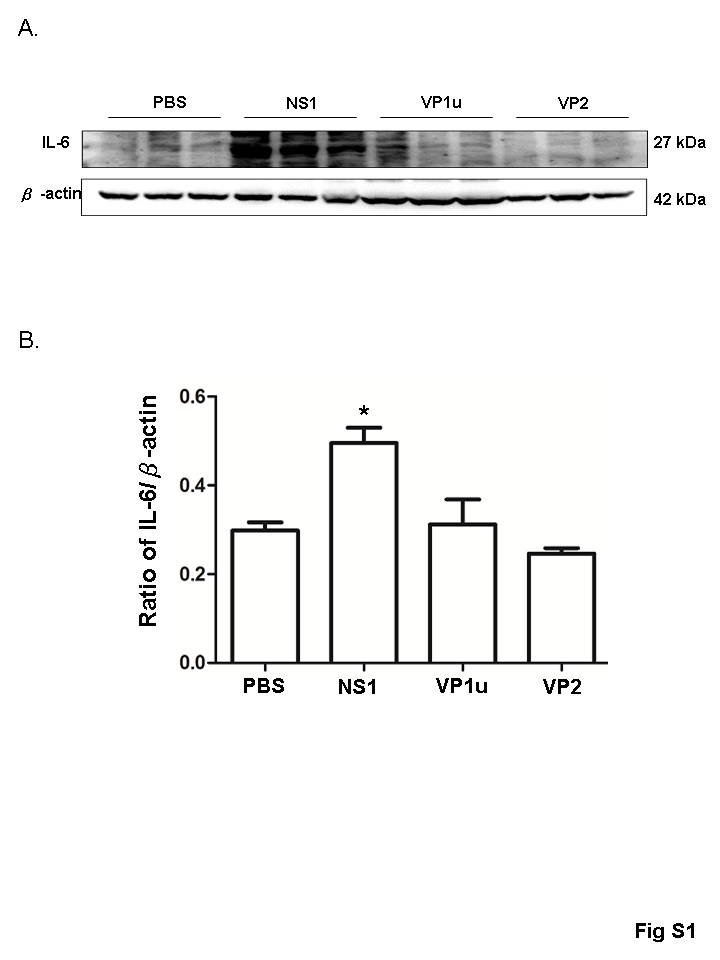

Supplement: Figure S1 — Expression of IL-6. Liver lysates obtained from the NZB/W F1 mice receiving PBS, NS1, VP1u or VP2 were probed with antibodies against (A) IL-6. Bars represent the relative protein quantification of (B) Sp1 on the basis of β-actin. Similar results were observed in three independent experiments, and * indicates the significant difference, P<0.05. (TIF) [file pone.0068393.s001.tif]
